# Long-Term Effectiveness of a Decision Support App (Pink Journey) for Women Considering Breast Reconstruction Surgery: Pilot Randomized Controlled Trial

**DOI:** 10.2196/31092

**Published:** 2021-12-10

**Authors:** Su-Ying Fang, Pin-Jun Lin, Yao-Lung Kuo

**Affiliations:** 1 Department of Nursing College of Medicine National Cheng Kung University Tainan Taiwan; 2 Department of Nursing DaYeh University Changhua Taiwan; 3 Department of Surgery College of Medicine National Cheng Kung University Tainan Taiwan; 4 Department of Surgery National Cheng Kung University Hospital Tainan Taiwan

**Keywords:** breast cancer, breast reconstruction surgery, decision aid, decision support, mHealth, app, women

## Abstract

**Background:**

Various kinds of breast reconstruction (BR) options, including implants and autologous, and surgery techniques, including traditional and endoscope assisted, can be used to perform surgery. All options have their own advantages and disadvantages. Women decide on an option depending on the values and preferences they emphasize. Lacking knowledge about BR or having decision difficulties during the treatment decision process makes women experience more decision regret, psychological distress, and poor body image. Delivering decision support with a values clarification exercise using eHealth approaches would be beneficial for patient outcomes.

**Objective:**

This study aims to examine the effects of a decision support app on decision-making quality and psychological morbidity for women considering BR surgery.

**Methods:**

This randomized controlled trial included women who were over 20 years of age and were newly diagnosed with breast cancer and candidates for mastectomy. Women having an option for breast conservation were excluded. After being referred from the outpatient physician, the women provided consent and completed the baseline assessment. Women allocated to the control group (CG) received usual care and were provided with a pamphlet with information about types of surgery and the advantages and disadvantages of different surgery types. Women allocated to the intervention group (IG) were given the same pamphlet and guided to use the Pink Journey app to support their decision. Then they were also prompted to discuss the opinions with their significant others. Finally, the decision-making process of using the app was printed out for women that they could take home. Decision conflict, anxiety, and depression were measured at baseline. At 1 week after the intervention (T1) and at 1 month (T2), 8 months (T3), and 12 months (T4) after surgery, the women completed decision conflict, decision regret, anxiety, depression, and body image scales. An intention-to-treat analysis was performed.

**Results:**

From February 2018 to July 2019, 96 women were randomly assigned to the CG (n=48) or the IG (n=48). Results revealed that body image distress declined significantly for the IG but increased for the CG. The interaction of time and group also reached significance, indicating a significant decrease in body image distress from baseline in the IG compared with the CG after the 12th month (T4) follow-up (β=–2.25, standard error=1.01, *P*=.027). However, there was no significant difference in decision conflict (*P*=.21-.87), decision regret (*P*=.44-.55), anxiety (*P*=.26-.33), and depression (*P*=.20-.75), indicating that the decrease in these outcomes in the IG was not greater than those in the CG.

**Conclusions:**

Although we found no effect on decision conflict, decision regret, anxiety, and depression, a decision aid that combines surgery information and values clarification can help women reduce their body image distress.

**Trial Registration:**

ClinicalTrials.gov NCT04190992; https://clinicaltrials.gov/ct2/show/NCT04190992

## Introduction

Breast cancer is the most prevalent cancer type in the world [1]. It is also the most common cancer among females in Taiwan [2]. Although breast conservative surgery (BCS) is now a standard treatment for early stage breast cancer, mastectomy rates in women eligible for BCS are increasing, with reports indicating that 35.5%-40% of women with breast cancer undergo mastectomy [3,4]. For women undergoing mastectomy, the change in appearance can lead to various types of psychological adjustment problems, including body image discomfort, psychological distress, anxiety, and depression [5-7]. Breast reconstruction (BR) has now become an option for women to restore their appearance. One cohort study revealed that the rates of BR increased from 11.6% in 1998 to 36.4% in 2011 (*P*<.001 for trend) [4].

BR can be performed immediately after a mastectomy or delayed according to each woman’s preferred timing after all required treatments have been completed [8]. Furthermore, various kinds of BR options (including implants and autologous) and surgical techniques (including traditional and endoscope assisted) can be used to perform the surgery [9]. All options have their own advantages and disadvantages. Women decide on an option depending on the values and preferences they emphasize [10]. Because of a new diagnosis and the nature of complex medical treatments involved, women feel stressed when making decisions related to surgery. Although a recent review study revealed that women are satisfied with their new breasts and reported low regret after receiving BR surgery [11], many felt surprised and perceived the reconstructed breasts to be unnatural, unreal, and unequal, and that the outcome was different from their original expectations before surgery [12,13]. Indeed, some women also reported high levels of decision regret after undergoing BR [14]. One recent study reported that patients undergoing BR preferred only a mastectomy that reflected a discordance with preferences [15]. Other studies documented the idea that if women lack BR knowledge or have decision difficulties during their treatment decision process, they experience more decision regret, psychological distress, and poor body image [16-18]. Helping women to make appropriate decision in accordance with their own values would be beneficial for their psychological well-being after surgery.

According to the Ottawa Decision Support Framework (ODSF), decision support needs to cover the provision of treatment/disease information, clarification of personal values, and assessment of support resources [19]. Furthermore, the decision aid (DA) following ODSF to support a treatment decision has to be validated to be helpful in improving knowledge, decreasing decision conflict, and increasing the consistency between the chosen option and personal values [20]. However, studies examining the effects of decision aid on helping women make BR surgery decision remain limited [21]. These studies found that the effect of intervention on decision conflict may occur within a short period, but the effect on decision regret may be delayed and occur much longer after an intervention. The effect of decision aid on other psychological indicators such as anxiety, depression, and body image was rare and needs to be further explored [21].

Computer-based DAs, including CD-ROM, computerized multimedia programs, and websites, were validated to perform better than paper-based DAs due to their potential for wide use by patients [22]. A recent review also documented that using electronically delivered decision support with a values clarification exercise would be beneficial to patient outcomes [23]. Given that smartphone devices and downloaded apps are more convenient than other devices with or without an internet connection [24], the aim of this study is to examine the effects of a decision support app on decision-related outcomes and psychological indicators including body image, depression, and anxiety for women considering BR surgery.

## Methods

### Study Design

This 1:1 randomized controlled parallel-arm trial with permuted block randomization that compares pamphlet + app with pamphlet alone was performed in Taiwan. The protocol was registered with ClinicalTrials.gov (NCT04190992), and the process of app development was published previously [25]. This study was approved by the Institution Review Board of National Cheng Kung University (B-ER-106-072) and was performed in accordance with the ethical principles of the Declaration of Helsinki. None of the data collected contained identifiable information; data were kept locked in the office of the first author (S-YF).

### Participants

Women were eligible for participation if they were (1) 20 years of age or older, (2) newly diagnosed with breast cancer and candidates for mastectomy, and (3) able to read and speak Taiwanese or Mandarin. Women were excluded if they had an option for BCS, reconstruction following a lumpectomy, reported active psychiatric illness, or severe cognitive illnesses that would prevent full participation. They were enrolled from February 2018 to July 2019 and completed their last follow-up in February 2021.

### Randomization, Blinding, and Procedure

Women were referred to the study by an outpatient physician. After signing informed consent, women completed the baseline assessment. Consenting women were randomized using online automated randomization software (Create a Randomization List [26]) to determine group allocation. Permutated block randomization (allocation ratio 1:1) was performed to maintain equal sample sizes. An independent research assistant generated the allocation sequence and prepared 136 numbered, opaque, sealed envelopes with assignments to be equally distributed between the 2 study groups. The researcher (S-YF) opened 1 envelope for each participant in the order in which she received a message or call from the interviewer (PJ-L) indicating that the participants were ready for randomization. The participants were not blinded to their allocation. At 1 week after intervention (T1) and at 1 month (T2), 8 months (T3), and 12 months (T4) after surgery, the women completed a follow-up questionnaire during their routine clinic visits.

### Intervention Versus Usual Care

**Figure 1:**
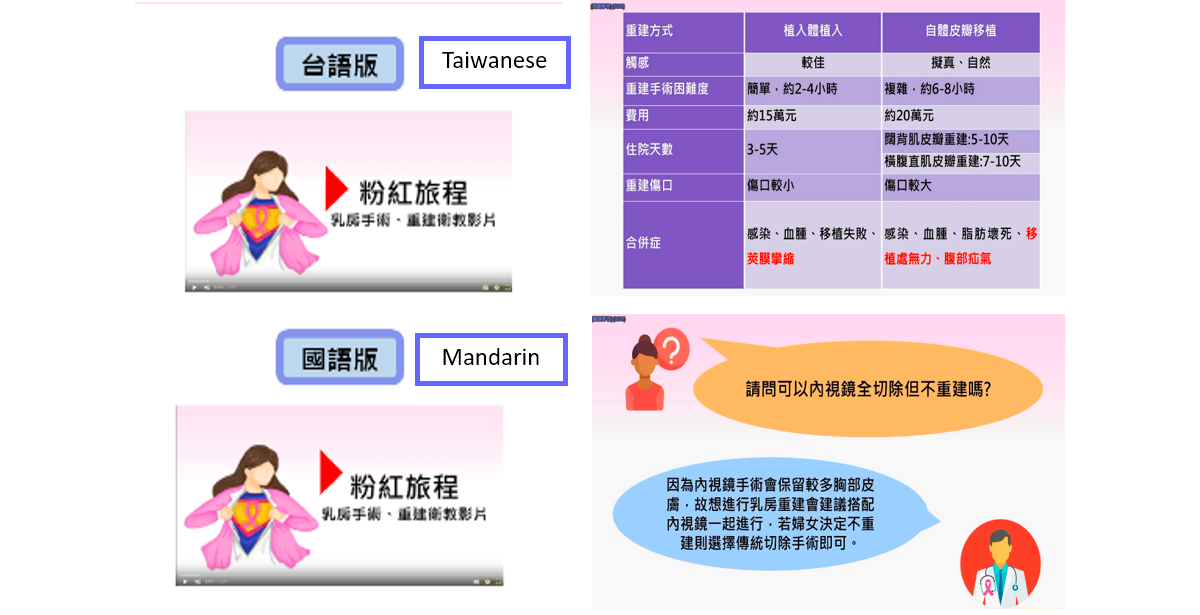
Two languages of the education video.

Women allocated to the control group (CG) received usual care from health care providers. They were also provided with a pamphlet with information about types of surgery, including mastectomy, implant-based BR, and autologous BR, and the advantages and disadvantages of different surgery types. Women allocated to the intervention group (IG) were given the same pamphlet and were further guided to use the Pink Journey app to support their decision [25]. Women first saw a video that is compatible with the content of the pamphlet and available in 2 languages (Chinese and Taiwanese), with selection depending on participant’s preference (Figure 1). Next, they were coached on how to use a values calcification exercise that elicited them to think about 10 possible factors that they were concerned about and to rank their concerns. They were then also prompted to discuss the opinions with their significant others. Finally, the decision-making process of using the app was printed out for women that they could take home. Detailed information about the Pink Journey app was published elsewhere [25].

### Instruments

Baseline data collection (T0) included demographic data and clinical data from the medical records of the patients. The following 5 instruments using a paper format were also administered at T0-T4 (ie, at baseline, 1 week, and 1, 8, and 12 months).

The Decision Conflict Scale (DCS) with 16 items developed by O’Connor [27] assesses the perception of uncertainty in information, values, or support for surgery options. The items were summed, divided by 16, and multiplied by 25. According to the user manual, scores below 25 were associated with more certainty of their decision; scores exceeding 37.5 indicated a greater feeling of uncertainty about their decision. This scale was also validated for Chinese women with surgery decisions related to breast cancer [28]. The Cronbach α coefficient for this scale in this study was .93.

The understanding of medical information was evaluated using the subscale of Involvement in the BR Decision-Making Process scale. This subscale with 6 items assesses perception of medical information about surgery and provides us information about women’s understanding of BR. The scale uses a 5-point Likert scale (1=*strongly disagree* to 5=*strongly agree*), where the higher the score, the greater the amount of information women believed they had obtained. This scale had good construct validity and good internal and test–retest reliability [18]. The Cronbach α coefficient for this scale in this study was .88.

The Decision Regret Scale (DRS) contains 5 items that assess distress or remorse after a surgery decision. The items were summed, divided by 5, and then multiplied by 25. This scale was also validated for Chinese women with surgery decisions related to breast cancer [28]. The Cronbach α coefficient for this scale in this study was .90.

The Body Image Scale (BIS) with 10 items was developed by Hopwood et al [29]. The scale uses a 4-point Likert scale (0=*not at all* to 3=*very much*), with total scores ranging from 0 to 30. Higher scores indicate greater body image distress. This scale has been widely used in numerous countries and in many languages in samples of patients with cancer [5]. The Cronbach α coefficient for this scale in this study was .92.

The Hospital Anxiety and Depression Scale (HADS) includes 14 items, of which 7 measure anxiety and 7 measure depression. All items are scored using a 0-3-point scale, with higher scores indicating more depressive symptoms. Cut-off scores are 8 and 11 to categorize the severity of anxiety and depressive symptoms, respectively. Values of over 8 indicate possible anxiety and depression, whereas values of 11 or above indicate probable anxiety and depression. The Chinese version of the scale has been widely used with good reliability and validity for women with breast cancer [5].

### Statistical Analysis

Descriptive statistics were used to describe baseline and background demographic data. The chi-square test for categorical variables and independent *t* test for continuous variables were used to examine homogeneity between groups and assess covariates. An intention-to-treat analysis was conducted using a mixed effects model analysis using SPSS (version 24; IBM, Inc.) with significance set at *P*<.05. A mixed effects model that included the study group, a categorical indicator of time, and the interaction between groups and times was generated after controlling for covariates (with a significant interaction indicating that compared with the CG, the intervention effects change over time). An autoregressive covariance structure analyzed changes among the time points and residual maximum likelihood to estimate the fixed effects. Missing data were not imputed due to low attrition rate 16/96 (17%), but restricted maximum likelihood estimation was used for data management.

## Results

### Study Flow and Participant Characteristics

**Figure 2:**
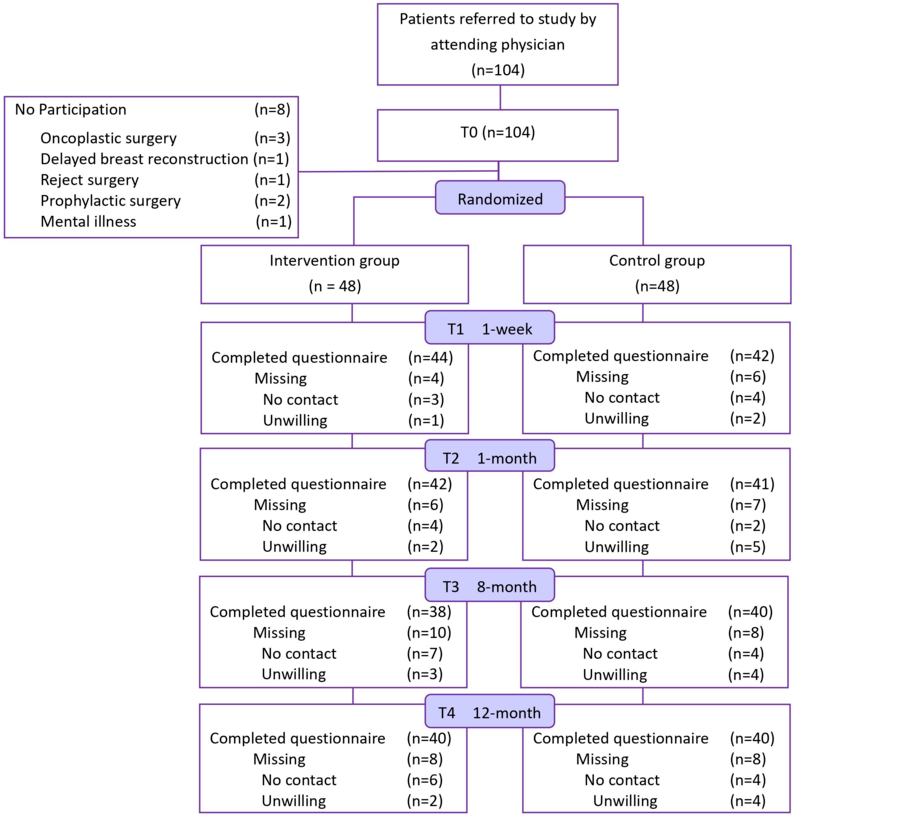
CONSORT (Consolidated Standards of Reporting Trials) diagram showing the flow of participants through each stage of the randomized controlled trial.

A total of 104 women were referred by a physician and completed the baseline questionnaires. Eight women dropped out after the pretest because they were rejected for surgery (n=1); scheduled for delayed (n=1), prophylactic (n=2), oncoplastic BR (n=3); or had a psychiatric disease (n=1; Figure 2). The remaining 96 women then were randomly assigned to either the CG or the IG. At T4, 72 women had provided complete data for each time point.

Among these women, 25 received neoadjuvant chemotherapy before surgery. A total of 38/96 (40%) women received chemotherapy, and 19/96 (20%) received radiotherapy following surgery. At baseline, there were no between-group differences in terms of demographic (Table 1) and disease-/treatment-related (Table 2) characteristics except that the women in the IG were younger than those in the CG (*P*=.01). In addition, women in both groups had similar preoperative appearance satisfaction (*P*=.15), anxiety (*P*=.09), and depression (*P*=.09) scores. Given the significant difference in age at diagnosis (*P*=.01) between women in the 2 groups, age may have played a role in BR decision and body image concerns, so the mixed effects analyses were adjusted for this variable.

**Table 1:** Demographic characteristics of the participants (N=96).

Characteristic		Total (N=96)	Intervention group (n=48)	Control group (n=48)	*P* value^a^		
**Age (years)**					*.011*		
	Mean (SD)	48.81 (8.22)	46.71 (8.19)	50.92 (7.77)			
	Range	27-71	27-71	32-68			
**Age groups (years), n (%)**							
	≤44	28 (29)	19 (40)	9 (19)			
	45-64	61 (64)	26 (54)	35 (73)			
	≥65	7 (7)	3 (6)	4 (8)			
**BMI (kg/m^2^)**					.66		
	Mean (SD)	22.68 (3.27)	22.53 (3.09)	22.83 (3.46)			
	Range	17.31-36.57	17.31-30.67	18.83-36.57			
**Education level (years), n (%)**						.32	
	<9	17 (18)	5 (10)	12 (25)			
	9-12	26 (27)	12 (25)	14 (29)			
	≧13	53 (55)	31 (65)	22 (46)			
Relationship status (% partner), n (%)		71 (74)	33 (69)	38 (79)	.25		
Employment status (% employed), n (%)		66 (69)	34 (71)	32 (66)	.66		
**Monthly household income (US $), n (%)**							.297
	Lower class (<1000)	19 (20)	7 (15)	12 (25)			
	Middle class (1001-1666)	27 (28)	11 (23)	16 (33)			
	Middle high (1667-3333)	24 (25)	14 (29)	10 (21)			
	High (>3333)	18 (19)	12 (25)	6 (13)			
	Unknown	8 (8)	4 (8)	4 (8)			
Private insurance, n (%)		80 (83)	42 (88)	38 (79)	.27		

^a^Statistical significance.

**Table 2:** Disease-related characteristics of the participants after surgery (N=96).

Characteristics			Total (N=96)		Intervention group (n=48)		Control group (n=48)		*P* value	
**Tumor size (cm), n (%)**										.63
	<1	11 (11)		5 (10)		6 (13)				
	1-3	49 (51)		22 (46)		27 (46)				
	>3	21 (22)		12 (25)		9 (19)				
	Missing	15 (16)		9 (19)		6 (13)				
**Breast cancer stage, n (%)**										.54
	0-I	45 (47)		23 (48)		22 (46)				
	II-III	34 (35)		15 (31)		19 (40)				
	Missing	17 (18)		10 (21)		7 (15)				
**Neoadjuvant chemotherapy, n (%)**										.96
	Yes	25 (26)		12 (25)		13 (27)				
	No	57 (59)		27 (56)		30 (63)				
	Missing	14 (15)		9 (19)		5 (10)				
**Chemotherapy, n (%)**										.73
	Yes	38 (40)		17 (35)		21 (44)				
	No	40 (42)		22 (46)		18 (38)				
	Missing	18 (19)		9 (19)		9 (19)				
**Radiotherapy, n (%)**										.80
	Yes	19 (20)		10 (21)		9 (19)				
	No	59 (61)		29 (60)		30 (63)				
	Missing	18 (19)		9 (19)		9 (19)				
**Surgical location, n (%)**										.59
	Unilateral	70 (73)		32 (67)		38 (79)				
	Bilateral	13 (14)		7 (15)		6 (14)				
	Missing	13 (14)		9 (19)		4 (8)				
**Lymphadenectomy, n (%)**										.55
	Sentinel lymph node biopsy	68 (71)		33 (69)		35 (73)				
	Axillary lymph node dissection	15 (16)		6 (13)		9 (19)				
	Missing	13 (14)		9 (19)		4 (8)				
**Active therapy 1 month after surgery, n (%)**										
	Chemotherapy	12 (13)		4 (8)		8 (17)		.25		
	Radiation therapy	4 (4)		3 (6)		1 (2)		.29		
**Active therapy 8 months after surgery, n (%)**										
	Chemotherapy	1 (1)		0 (0)		1 (2)		.34		
	Radiation therapy	2 (2)		0 (0)		2 (4)		.16		
**Active therapy 12 months after surgery, n (%)**										
	Chemotherapy	2 (2)		0 (0)		2 (4)		.16		
	Radiation therapy	1 (1)		0 (0)		1 (2)		.32		
**Self-evaluation on body appearance, mean (SD)**										
	The difference in appearance between reality and ideality (range 0-10)	4.22 (2.80)		4.27 (2.89)		4.17 (2.73)		.86		
	The importance of appearance in life (range 0-10)	5.53 (2.58)		5.35 (2.61)		5.71 (2.55)		.50		

### Decision-Making Quality

Table 3 summarizes the findings of decision-making quality outcomes. Overall, this sample reported an average of DCS score that was higher than the cut-off of 37.5. However, decision conflict declined significantly after the 1-week follow-up for both groups. Furthermore, the interaction of time and group did not reach significance (β=–2.79, standard error [SE]=3.72, *P*=.46), indicating that the decreasing of DCS score in the IG was not greater than that in the CG (Table 3).

The amount of medical information related to BR at 1 week after consultation did not differ between the IG and CG (*P*=.13), which indicates that women in both groups perceived a similar understanding level related to medical information, whether using just a pamphlet or combined with app.

Decision regret did not differ between groups at 1 month (*P*=.51), 8 months (*P*=.66), or 12 months (*P*=.61), and the interaction of time and group also did not reach significance (*P*=.44-.55).

**Table 3:** Between-group differences using the mixed effect model of decision-making quality.

Outcome measure					Intervention group, mean (SD)		Control group, mean (SD)		*P* value		β		Standard error			95% CI			*P* value	
**T0 (Baseline)^a^**																				
	**Decision Conflict Scale (0-100)**																			
		Total		38.28 (17.22)		42.90 (21.52)		.25												
		Informed		48.26 (21.54)		51.39 (26.71)		.53												
		Values		51.22 (23.19)		56.94 (27.52)		.27												
		Support		25.52 (20.80)		31.25 (24.40)		.22												
		Uncertainty		37.67 (20.56)		41.67 (28.71)		.44												
		Effective decision		31.12 (20.20)		35.68 (25.78)		.34												
**T1 (1-week postconsultation)^b^**																				
	**Decision Conflict Scale (0-100)**																			
			Total	19.35 (15.68)		20.87 (16.65)		.67		2.79		3.72			–4.61 to 10.19			.46		
			Informed	17.05 (19.44)		21.43 (21.00)		.32		–1.79		4.86			–11.44 to 7.87			.71		
			Values	20.45 (18.10)		24.80 (24.45)		.35		0.90		5.45			–9.93 to 11.73			.87		
			Support	14.39 (18.71)		14.09 (16.20)		.94		5.87		5.00			–4.06 to 15.80			.24		
			Uncertainty	26.70 (19.07)		23.81 (20.71)		.50		6.66		5.22			–3.72 to 17.04			.21		
			Effective decision	18.47 (16.34)		20.39 (16.51)		.60		2.59		4.94			–7.22 to 12.40			.60		
			Amount of medical information	20.25 (4.91)		21.76 (4.19)		.13												
**T2 (1 week after consultation)^c^**																				
	Decision Regret Scale (0-100)				19.52 (15.53)		21.95 (18.00)		.51											
**T3 (8 months after surgery)^d^**																				
	Decision Regret Scale (0-100)				21.84 (23.38)		19.63 (20.83)		.66		3.38		5.70			–7.84 to 14.60			.55	
**T4 (12 months after surgery)^e^**																				
	Decision Regret Scale (0-100)				21.63 (23.95)		19.25 (7.45)		.61		3.69		4.73			–5.66 to 13.04			.44	

^a^n=48 in the intervention and control group, respectively.

^b^n=44 and 42 in the intervention and control group, respectively.

^c^n=42 and 41 in the intervention and control group, respectively.

^d^n=38 and 40 in the intervention and control group, respectively.

^e^n=40 in the intervention and control group, respectively.

### Psychological Indicators

#### Body Image and Appearance Satisfaction

Table 4 summarizes the findings of psychological outcomes. Body image distress declined significantly over time for both groups. The interaction of time and group also reached significance, indicating a significant decrease in body image distress from the baseline in the IG compared with the CG after the 12-month (T4) follow-up (β=–2.25, SE=1.01, *P*=.027).

There was also a tendency toward an improvement in appearance satisfaction over time in both groups. The interaction of time and group reached significance from T3 (β=1.15, SE=0.57, *P*=.045) to T4 (β=1.17, SE=0.54, *P*=.031), which indicated that the improvement in appearance satisfaction from baseline in the IG compared with the CG was significant after the 8-month follow-up (*P*=.031-.045).

**Table 4:** Between-group differences using the mixed effect model of psychological distress.

Outcome measure				Intervention group, mean (SD)		Control group, mean (SD)		*P* value		β		Standard error			95% CI			*P* value
**T0 (Baseline)^a^**																		
	**HADS^b^ (0-21)**																	
		Anxiety	8.38 (4.55)		9.98 (4.66)		.09											
		Depression	6.12 (3.76)		7.48 (3.85)		.09											
		Body satisfaction (0-10)	5.46 (2.16)		6.08 (2.08)		.15											
**T2 (1 month after surgery)^c^**																		
	**HADS (0-21)**																	
		Anxiety	5.64 (4.33)		5.85 (3.84)		.82		1.23		1.09			–0.90 to 3.37			.256	
		Depression	5.64 (3.52)		5.93 (4.08)		.74		0.93		1.04			–1.11 to 2.98			.370	
		Body satisfaction (0-10)	6.64 (1.76)		6.71 (2.33)		.89		0.44		0.58			–0.70 to 1.57			.447	
		Body Image Distress (0-30)	6.38 (5.55)		6.49 (6.09)		.93											
**T3 (8 months after surgery)^d^**																		
	**HADS (0-21)**																	
		Anxiety	4.66 (3.68)		4.88 (3.72)		.80		0.96		0.99		–0.99 to 2.90			.334		
		Depression	5.29 (4.13)		5.03 (3.82)		.77		1.23		0.96		–0.66 to 3.11			.202		
		Body satisfaction (0-10)	7.08 (1.94)		6.78 (2.38)		.54		1.15		0.57		0.25 to 2.27			*.045* ^e^		
		Body Image Distress (0-30)	6.08 (6.02)		6.93 (6.15)		.54		–0.91		1.32		–3.52 to 1.69			.490		
**T4 (12 months after surgery)^f^**																		
	**HADS (0-21)**																	
		Anxiety	4.25 (3.70)		4.88 (3.52)		.44		0.86		0.78		–0.68 to 2.39			.273		
		Depression	4.03 (3.93)		5.03 (3.71)		.25		0.24		0.77		–1.27 to 1.76			.752		
		Body satisfaction (0-10)	7.98 (1.46)		7.03 (2.20)		.41		1.17		0.54		0.11 to 2.24			*.031* ^e^		
		Body Image Distress (0-30)	4.35 (0.69)		7.11 (1.12)		.05		–2.25		1.01		–4.24 to –0.26			.027		

^a^n=48 in each group.

^b^HADS: Hospital Anxiety and Depression Scale.

^c^n=42 and 41 in the intervention and control group, respectively.

^d^n=38 and 40 in the intervention and control group, respectively.

^e^Statistical significance.

^f^n=40 and 40 in the intervention and control group, respectively.

#### Anxiety and Depression

The HADS anxiety scores 1 (*P*=.82), 8 (*P*=.80), and 12 months (*P*=.44) after surgery did not differ between groups. The HADS depression scores 1 (*P*=.74), 8 (*P*=.77), and 12 months (*P*=.25) after surgery also did not differ between groups. There was a tendency toward a decrease in depression and anxiety over time in both groups. However, the interaction of time and group did not reach significance (Table 4).

### Choice of Surgery

Choice of surgery differed between the IG and CG. Overall, 56% (27/48) and 46% (22/48) opted for mastectomy plus immediate reconstruction in the IG and CG, respectively (*P*=.05). Moreover, a majority selected implanted-based BR, which did not differ between groups (Table 5).

**Table 5:** Comparison of surgical decision between groups.

Decision		Total (n=96)	Intervention group (n=48)	Control group (n=48)	*P* value	
**Breast reconstruction**						*.048* ^a^
	Yes, n (%)	49 (51)	27 (56)	22 (46)		
	No, n (%)	36 (38)	14 (29)	22 (46)		
	Missing, n (%)	11 (11)	7 (15)	4 (8)		
**Breast reconstruction type**						.160
	Implant based, n/N (%)	46/49 (94)	26/27 (96)	20/22 (91)		
	Autologous, n/N (%)	3/49 (6)	1/27 (4)	2/22 (9)		

^a^Statistical significance.

## Discussion

### Decision-Making Quality

This study evaluated the effects of app-based DA on women’s decision quality and postoperative psychological morbidity regarding BR. Women who received the app-based DA reported a similar decline in decision conflict 1 week after consultation compared with women only receiving standard care with a pamphlet. This result is consistent with a study using an interactive web-based training program [30], but is in contrast to previous studies that revealed that breast cancer treatment DA reduced decision conflict to higher levels compared with a standard booklet after consultation [31-33]. In Luan et al’s study [33], only postconsultation score was compared, and it was not clear whether the decreasing level of decision conflict between baseline and postconsultation was significant. In Sherman et al’s study [32], decision conflict was measured 1 month after baseline, so women who completed surgery or not may bias this outcome. In Lam et al’s study [31], women in the CG only received standard information without a take-home booklet, which is different from our study, as we provided a take-home pamphlet also to the CG. Low statistical power may exist due to small differences in the treatments designed in our study compared with those in Lam et al’s study [31]. Manne et al’s study [30] was similar to our study by providing a pamphlet to the CG, and it revealed no significant change in decision conflict over a short period. The amount of medical information women received was not significant between groups in our study, which suggests that improving knowledge about BR may decrease women’s decision conflict over a short period, but its long-term effects should be examined.

There was no difference in decision regret 1, 8, and 12 months after surgery between the 2 groups. Previous studies have revealed lower regret in the IG; however, these studies only measured 1 period and without a follow-up for over 6 months [32,33]. Lam et al’ study [31] revealed decreased decision regret 4 and 10 months after surgery. This may be because most participants in their study also had the option to undergo BCS [31]. By contrast, in our study, mastectomy was necessary and there was no option to choose BCS, and this may have contributed to the nonsignificant result in this study.

### Psychological Distress

There was no significant difference in appearance satisfaction and body image distress 1 month after surgery between the IG and CG. However, at both 8 and 12 months after surgery, women in the CG reported significantly better appearance and lower body image distress. A limited number of studies examined the effect of DA on body image distress. Using BREAST-Q assessment, Politi et al [34] found that satisfaction with breasts score in the DA group was slightly higher than that in the CG. Luan et al [33] reported that sum scores of sexual well-being satisfaction, satisfaction with breasts, outcome, and care in the DA group were more likely to be higher than those in the CG; however, no statistical significance was revealed in the aforesaid studies. These studies evaluated patients’ feedback only over a short period, but our study highlights the importance of accessing both short- and long-term impacts of BR surgery on body image distress. Our study is the first to demonstrate significantly lower body image distress among women in the IG compared with the CG 12 months after surgery. Supporting our hypothesis, adding a values clarification exercise in DA may have helped women to create more realistic expectations about outcomes after BR, decreasing the sense of loss and reducing their body image distress.

Our analysis demonstrated that providing information using a paper or digital format in combination with values clarification did not increase anxiety for either group. This result is consistent with studies with a similar design that provided a pamphlet to the CG with short [30] and long follow-ups [33]. No significant difference between groups regarding depression was also consistent with a recent study [33]. Body image distress is associated with depression. In our study, body image distress significantly improved in the IG compared with the CG at the 12-month follow-up, suggesting that continuous follow-up to clarify the effect of DA is necessary.

Given the characteristics of universal national health insurance and the convenient geographical environment in Taiwan, losing women to follow-ups is usually because they tend to search for a second opinion in other hospitals. Although the attrition rate is not higher (17%), we do acknowledge that this was a pilot randomized controlled trial and a relatively small sample size may underestimate the effects of this study. Second, because breast surgeons are generally familiar with only 1 surgical technique, inclusion of a single breast surgeon in 1 medical center limits this study’s generalization. Lastly, our study used an amount of medical information to evaluate women’s perception of their understanding of BR knowledge and only assessed it 1 week after intervention without a preintervention assessment. This limited us from comparing the changes in knowledge between groups.

### Conclusions

This was the first trial to examine the long-term effects of an app-based DA on both decision-making quality and psychological morbidity for women only having the option for mastectomy. It demonstrates that DA designed with values clarification exercises can reduce similar decision conflict and depression without increasing anxiety over time compared with only receiving a pamphlet. It also further supports that using values clarification exercises can help women reduce their body image distress and increase body appearance satisfaction. Because low monitors who were highly anxious about detailed information had a greater likelihood of experiencing regret [14], future DA trials should also consider monitoring coping style and DA design that could be tailored to each women’s needs. In addition, DA with a value clarification exercise that considers personal value and shows an effect on body image distress supports the utilization of personalized treatments, such as nanomedicine and immune therapy. These therapies specify women’s tumor characteristics to increase therapeutic effect but with fewer side effects, empathizing that precision care for women with breast cancer will become a trend in the future [35-37].

## Appendix

Multimedia Appendix 1 CONSORT-eHEALTH checklist (V 1.6.1).

## Abbreviations

BCS: breast conservative surgery
BIS: Body Image Scale
BR: breast reconstruction
CG: control group
DA: decision aid
DCS: Decision Conflict Scale
DRS: Decision Regret Scale
HADS: Hospital Anxiety and Depression Scale.
IG: intervention group
ODSF: Ottawa Decision Support Framework
SE: standard error
